# Apoptosis of Endothelial Cells Contributes to Brain Vessel Pruning of Zebrafish During Development

**DOI:** 10.3389/fnmol.2018.00222

**Published:** 2018-06-28

**Authors:** Yu Zhang, Bing Xu, Qi Chen, Yong Yan, Jiulin Du, Xufei Du

**Affiliations:** ^1^Institute of Neuroscience, State Key Laboratory of Neuroscience, Center for Excellence in Brain Science and Intelligence Technology, Chinese Academy of Sciences, Shanghai, China; ^2^School of Future Technology, University of Chinese Academy of Sciences, Beijing, China; ^3^School of Life Science and Technology, ShanghaiTech University, Shanghai, China

**Keywords:** apoptosis, endothelial cells, vessel pruning, microglia, zebrafish

## Abstract

During development, immature blood vessel networks remodel to form a simplified and efficient vasculature to meet the demand for oxygen and nutrients, and this remodeling process is mainly achieved via the pruning of existing vessels. It has already known that the migration of vascular endothelial cells (ECs) is one of the mechanisms underlying vessel pruning. However, the role of EC apoptosis in vessel pruning remains under debate, especially in the brain. Here, we reported that EC apoptosis makes a significant contribution to vessel pruning in the brain of larval zebrafish. Using *in vivo* long-term time-lapse confocal imaging of the brain vasculature in zebrafish larvae, we found that EC apoptosis was always accompanied with brain vessel pruning and about 15% of vessel pruning events were resulted from EC apoptosis. In comparison with brain vessels undergoing EC migration-associated pruning, EC apoptosis-accompanied pruned vessels were longer and showed higher probability that the nuclei of neighboring vessels’ ECs occupied their both ends. Furthermore, we found that microglia were responsible for the clearance of apoptotic ECs accompanying vessel pruning, though microglia themselves were dispensable for the occurrence of vessel pruning. Thus, our study demonstrates that EC apoptosis contributes to vessel pruning in the brain during development in a microglial cell-independent manner.

## Introduction

During development, highly ramified immature blood vascular networks undergo extensive remodeling, including refined vessel pruning of selected vessel branches and complete regression of vascular networks, to form an efficient mature vasculature to meet their physiological functions (Adams and Alitalo, 2007; Herbert and Stainier, 2011; Korn and Augustin, 2015; Betz et al., 2016). It is generally thought that vessel pruning is mainly achieved through blood flow fluctuation-induced lateral migration of endothelial cells (ECs) to adjacent unpruned vascular segments (Chen et al., 2012; Kochhan et al., 2013; Franco et al., 2015; Lenard et al., 2015). However, EC apoptosis has been found during the pruning of the cranial division of internal carotid artery (CrDI) in zebrafish (Kochhan et al., 2013) and of the retinal vasculature in mice (Franco et al., 2015). In particular, macrophage-induced EC apoptosis is responsible for the complete regression of the hyaloid vasculature in developing eyes (Lobov et al., 2005). Therefore, the contribution of EC apoptosis to vessel pruning, especially in the brain vasculature, remains unclear.

Microglia, the resident immune cells in the central nervous system (CNS), are specialized macrophages that play crucial roles in mediating immune-related functions (Nayak et al., 2014). Besides being immune mediators, microglia are emerging as important contributors to normal CNS development and function (Paolicelli et al., 2011; Li et al., 2012; Wu et al., 2015). Interestingly, microglia are also shown to be important for the behaviors of vascular tip cells (Fantin et al., 2010; Tammela et al., 2011; Arnold and Betsholtz, 2013), the repair of brain vascular rupture (Liu et al., 2016), and the closure of injured blood-brain barrier (Lou et al., 2016). However, it is still unknown whether microglia participate in brain vascular remodeling during development.

To address whether EC apoptosis and microglia are involved in brain vessel pruning, we performed *in vivo* long-term time-lapse confocal imaging of the brain vasculature in larval zebrafish, and first found that about 15% of brain vessel pruning events were accompanied by EC apoptosis. Furthermore, we demonstrated that microglia were unnecessary for brain vessel pruning but responsible for the clearance of pruned vessel-associated apoptotic ECs. Thus, our findings reveal that microglial cell-independent EC apoptosis is involved in brain vessel pruning of larval zebrafish.

## Materials and Methods

### Zebrafish Husbandry

Adult zebrafish (*Dario rerio*) were maintained in an automatic fish housing system at 28°C following standard protocols (Chen et al., 2012). The *Tg(kdrl:EGFP)s843* (Jin et al., 2005), *Tg(fli1a.ep:DsRedEx)um13* (Covassin et al., 2009), *Tg(fli1a:nEGFP)y7* (Roman et al., 2002) and *Tg(coro1a:DsRedEx)hkz011t* (Li et al., 2012) zebrafish lines were described previously. Zebrafish embryos were raised under a 14 h–10 h light-dark cycle in 10% Hanks’ solution that consisted of 140 mM NaCl, 5.4 mM KCl, 0.25 mM Na_2_HPO_4_, 0.44 mM KH_2_PO_4_, 1.3 mM CaCl_2_, 1.0 mM MgSO_4_ and 4.2 mM NaHCO_3_ (pH = 7.2). The 0.003% 1-phenyl-2-thiourea (PTU; Sigma, P7629) was added into the Hanks’ solution to prevent pigment formation of zebrafish embryos. The zebrafish chorion was removed at 1 day post-fertilization (dpf) with the treatment of 2 mg/ml pronase (Calbiochem, 53702), which was diluted in the Hanks’ solution. All animal use and handling procedures were approved by the Institute of Neuroscience, Chinese Academy of Sciences.

### *In Vivo* Confocal Imaging

Imaging was performed on 3–3.5 dpf zebrafish larvae at room temperature (26–28°C). Larvae were embedded in 1% low-melting agarose (Sigma) for imaging without anesthetics. Imaging was carried out under a 40× water immersion objective (N.A., 0.80) with an Olympus Fluoview 1000 confocal microscope (Tokyo, Japan). The *z*-step of imaging was 3 μm. To trace the fate of each vessel segment during development, long-term time-lapse imaging of the brain vasculature of the same larvae was performed with 1-h interval.

### Image Analysis

All the images were analyzed by ImageJ (NIH). The pruned vessels were quantified in the whole zebrafish brain. For the quantification of vessel pruning, the vessel segment which initially displayed lumen morphology but with a collapsed shape and disappeared completely later was counted as a pruned vessel. For the quantification of EC apoptosis, the ECs exhibiting typical apoptotic features, such as plasma membrane blebbing and formation of apoptotic bodies, were counted as apoptotic ECs. For the quantification of microglial density, we counted the number of microglia in the whole brain region of the microglial transgenic zebrafish *Tg(coro1a:DsRedEx)* microinjected with control or *pu.1* MO. The microglial engulfment of apoptotic ECs was defined as a process that the microglia migrated to and engulfed the apoptotic ECs and then the apoptotic ECs faded away.

### Morpholino Oligonucleotides and Microinjection

Morpholino oligonucleotides (MOs) were purchased from Gene Tools (Philomath, OR, USA). Lyophilized MOs were dissolved in nuclease-free water. The 4 ng *pu.1* MO (5′-GATATACTGATACTCCATTGGTGGT-3′) or equivalent control MO (5′-CCTCTTACCTCAGTTACAATTTATA-3′) were microinjected into one-cell-stage zebrafish embryos.

### DAPI Injection

The DAPI (10 nl, 1 mg/ml) was microinjected into the blood circulation system of 3-dpf *Tg(kdrl:EGFP)* larvae through the common cardinal vein. Immediately after the injection, time-lapse imaging was performed to trace pruned vessels with apoptotic ECs.

### Immunofluorescence

Whole zebrafish embryos at 3 dpf were fixed in 4% paraformaldehyde for 2 h at room temperature, washed twice in PBS, and then incubated in a blocking solution (5% normal goat serum in PBS 0.2% Triton) overnight at 4°C. The embryos were then incubated in the blocking solution with a rabbit anti-Caspase-3 primary antibody (1:500, ab13847) for 72 h at 4°C. After washing with PBS 0.2% Triton, the embryos were incubated in the blocking solution with a secondary antibody (1:500) and DAPI (1:1000) for 48 h at 4°C. After washing with PBS 0.2% Triton, images were taken with an Olympus Fluoview 1000 confocal microscope (Tokyo, Japan). The dying ECs showing overlapped anti-Caspase-3 staining and DAPI staining are considered as the apoptotic ECs.

### Statistical Analysis

Statistical analyses were performed by using GraphPad Prism v6.0 software. The significance of the difference between two groups was determined by using two-tailed unpaired Student’s *t*-test. Data were represented as mean ± SEM, and *p* < 0.05 was considered to be statistically significant.

## Results

### Brain Vessel Pruning Is Accompanied With EC Apoptosis in Larval Zebrafish

**Figure 1:**
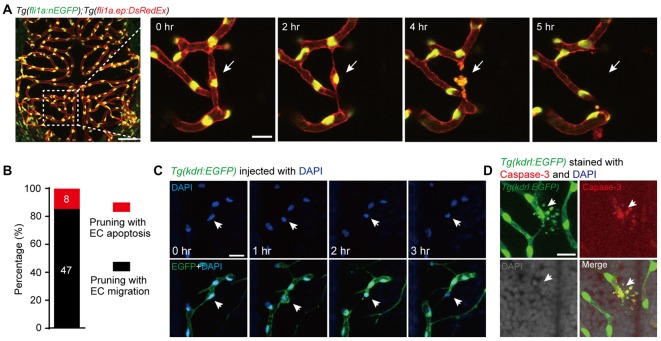
Vessel pruning is accompanied with endothelial cell (EC) apoptosis in the brain of larval zebrafish. **(A)** *In vivo* time-lapse confocal images showing that an EC (arrows) underwent apoptosis on a pruned brain vessel in a *Tg(fli1a:nEGFP);Tg(fli1a.ep:DsRedEx)* larva at 3–3.5 days post-fertilization (dpf), in which EC nuclei were labeled by both enhanced green fluorescent protein (EGFP) and DsRed (yellow). Left, projected confocal image of the whole brain vasculature; Right, time-lapse confocal images of the dashed outlined area in the left. **(B)** Summary of the percentages of EC apoptosis- and migration-accompanied brain vessel pruning (*n* = 17 larvae). **(C)** *In vivo* time-lapse confocal images showing that an EC (arrows) underwent apoptosis on a brain pruned vessel in a DAPI-injected *Tg(kdrl:EGFP)* larva at 3–3.5 dpf, in which EC nuclei were labeled by DAPI (blue). **(D)** Immunofluorescence images showing that, on a pruned vessel, an EC with typical apoptotic morphology (arrows) expressed Caspase-3. The numbers on the bars **(B)** represent the number of pruned vessels examined. Scales: 50 μm (left in **(A)**, 15 μm (right in **(A)**, 50 μm **(C)** and 15 μm **(D)**.

To examine whether the apoptosis of ECs contributes to brain vessel pruning, we monitored the development of the brain vasculature in individual larval zebrafish with 1-h interval during 3–3.5 dpf, when most brain vessel pruning events occur (Chen et al., 2012). The brain vasculature was visualized by using the double transgenic line *Tg(fli1a:nEGFP);Tg(fli1a.ep:DsRedEx)*, in which the nucleus and cellular morphology of ECs were labeled by enhanced green fluorescent protein (EGFP) and DsRed, respectively (Chen et al., 2012). Besides the previously reported EC lateral migration-associated vessel pruning, we also found EC death-accompanied vessel pruning, during which the dying EC showed typical morphological features of cell apoptosis, including plasma membrane blebbing and formation of apoptotic bodies (Figure 1A). In the total of 55 vessel pruning events observed in 17 larvae, 15% of them (8 out of 55) were accompanied with EC apoptosis (Figure 1B). Furthermore, by microinjection of DAPI into the circulation system of *Tg(kdrl:EGFP)* larvae to label the nucleus of ECs, we found that the nucleus of apoptotic ECs condensed and fragmented during vessel pruning (Figure 1C).

To demonstrate that the dying ECs with typical apoptotic morphology during vessel pruning are indeed undergoing apoptosis, we first screened out the vessel pruning event accompanied with dying ECs by *in vivo* imaging, and then fixed the embryo immediately to examine the expression of the active Caspase-3, a marker of cell apoptosis (Franco et al., 2015). We found that the dying EC indeed expressed active Caspase-3 and showed nuclear condensation and formation of apoptotic bodies, indicating the existence of EC apoptosis (Figure 1D). Taken together, these results demonstrate that EC apoptosis is indeed involved in the vessel pruning of the zebrafish brain vasculature during development.

### EC Apoptosis-Accompanied Pruned Vessels Are Longer and Show Higher Probability That Adjacent Vessels’ EC Nuclei Occupy Their Both Ends

**Figure 2:**
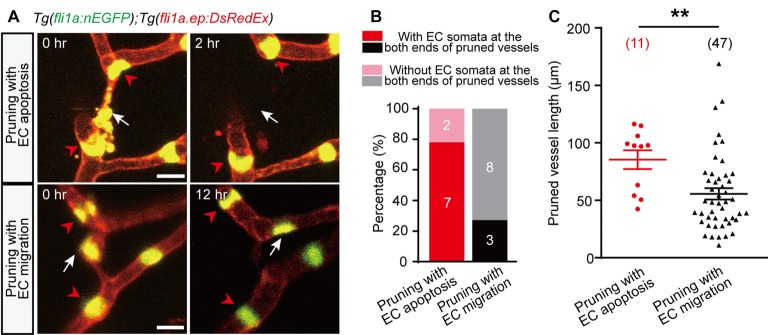
Characterization of EC apoptosis-accompanied pruned vessels. **(A)** *In vivo* time-lapse confocal images showing the relative spatial locations of EC nuclei in pruned and adjacent brain vessels. Top: for an EC apoptosis-accompanied pruned brain vessel, the nuclei of two neighboring vessels’ ECs (red arrowheads) located at the both ends of the pruned vessel, respectively (white arrow). Bottom: for an EC migration-accompanied pruned brain vessel, the nuclei of neighboring vessels’ ECs (red arrowheads) did not occupy the ends of the pruned vessel (white arrow). **(B)** Summary of data showing that EC apoptosis-accompanied pruning vessels have a higher probability that neighboring vessels’ EC nuclei occupy their both ends (7 out of 9, *n* = 7 larvae) than EC migration-accompanied pruning vessels (3 out of 11, *n* = 6 larvae). **(C)** Length of pruned vessels with EC apoptosis (*n* = 9 larvae) or EC migration (*n* = 13 larvae). The numbers on the bars **(B)** or in the brackets **(C)** represent the number of pruned vessels examined. Data are shown as mean ± SEM. **p < 0.01 (two-tailed unpaired Student’s *t*-test). Scale bar: 10 μm **(A)**.

Compared with pruned vessels with EC lateral migration, we found that EC apoptosis-accompanied pruned vessels showed a higher probability that the nuclei of neighboring vessels’ ECs located near the both ends of the pruned vessels (Figures 2A,B; pruning with EC apoptosis, 7 out of 9; pruning with EC migration, 3 out of 11). Moreover, EC apoptosis-accompanied pruned vessels were much longer (Figure 2C; pruning with EC apoptosis, 85 ± 8 μm; pruning with EC migration, 56 ± 5 μm; *p* < 0.01, two-tailed unpaired Student’s *t*-test). These results suggest that the vessel segments with longer length and occupied by the nuclei of neighboring vessels’ ECs at their both ends are more likely to be pruned via EC apoptosis rather than EC migration.

### Microglia Engulf Apoptotic ECs but Are Dispensable for Vessel Pruning

**Figure 3:**
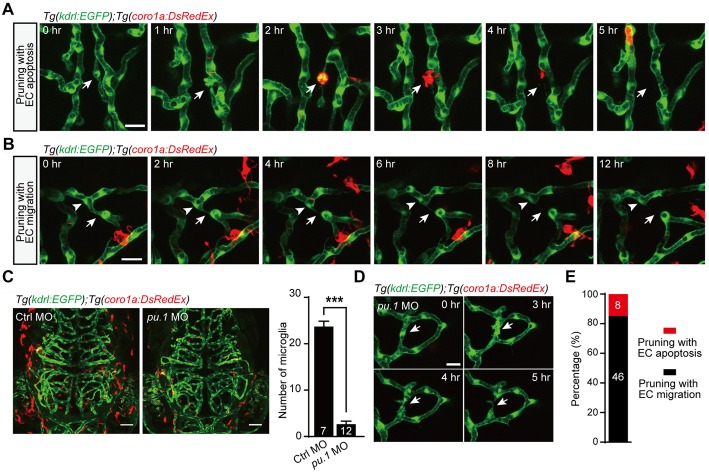
Role of microglia in EC apoptosis-accompanied brain vessel pruning. **(A)** *In vivo* time-lapse confocal images showing that, during EC apoptosis-accompanied vessel pruning, a microglial cell (red) migrated to, engulfed and cleaned the apoptotic EC (white arrow). The *Tg(coro1a:DsRedEx);Tg(kdrl:EGFP)* larvae at 3–3.5 dpf were used. **(B)** *In vivo* time-lapse confocal images showing that, during EC migration-accompanied vessel pruning (white arrow), there was no obvious interaction between microglia (red) and the migrating EC (white arrowhead). **(C)** Representative projected confocal images (left) and summary data (right) showing that knockdown of *pu.1* significantly diminished the number of microglia (red) in the brain at 3 dpf. **(D)** *In vivo* time-lapse confocal images showing that EC apoptosis-accompanied brain vessel pruning (arrow) still occurred in *pu.1* morphants. **(E)** Summary of the percentages of EC apoptosis- and migration-accompanied brain vessel pruning from *pu.1* MO-injected *Tg(coro1a:DsRedEx);Tg(kdrl:EGFP)* (*n* = 30 larvae). The numbers on the bars represent the number of animals **(C)** or pruned vessels **(E)** examined. Data are shown as mean ± SEM. ***p < 0.001 (two-tailed unpaired Student’s *t*-test). Scales: 20 μm **(A,B)**, 50 μm **(C)** and 15 μm **(D)**.

As previous studies showed that macrophage-dependent EC apoptosis is responsible for the complete regression of the hyaloid vessel network (Kochhan et al., 2013; Franco et al., 2015), we hypothesized that microglia may be involved in EC apoptosis-accompanied brain vessel pruning. Thus, we performed *in vivo* time-lapse confocal imaging of *Tg(coro1a:DsRedEx);Tg(kdrl:EGFP)* zebrafish larvae, in which DsRed and EGFP were expressed in microglia and ECs, respectively. Interestingly, we found that after the EC on the pruning vessel went into apoptosis, a microglial cell always migrated to and engulfed the apoptotic EC, followed by the completion of vessel pruning (Figure 3A). Whereas, in the EC lateral migration-accompanied vessel pruning, we did not find the interaction between microglia and pruned vessels (Figure 3B). The fact that the migration of microglia toward pruning vessel is after the EC apoptosis suggests that EC apoptosis is microglial cell-independent. However, microglia contribute to the clearance of the apoptotic ECs.

To further examine whether microglia are essential for EC apoptosis-accompanied vessel pruning, we downregulated the expression of the *pu.1* transcription factor, which is required for macrophage differentiation, through *pu.1* morpholino oligonucleotide (MO; Chen et al., 2012). In the *pu.1* MO-injected *Tg(coro1a:DsRedEx);Tg(kdrl:EGFP)* larvae, microglia were largely diminished (Figure 3C; *p* < 0.001, two-tailed unpaired Student’s *t*-test). However, in accordance with in normal larvae (see Figure 1B), the percentage of EC apoptosis-accompanied brain vessel pruning in microglial cell-deficient fish was also about 15% (Figures 3D,E, 8 out of 54). These results indicate that the occurrence of EC apoptosis-accompanied brain vessel pruning is microglial cell-independent, but microglia are responsible for the clearance of apoptotic ECs.

## Discussion

Taking advantage of *in vivo* time-lapse imaging on larval zebrafish, in this study we identified, to our knowledge, for the first time that EC apoptosis makes a significant contribution to brain vessel pruning during development in a microglial cell-independent manner. Although unnecessary for EC apoptosis-accompanied vessel pruning, microglia can engulf and clear apoptotic ECs, a behavior that may be important for protecting the brain microenvironment from inflammation.

A previous study showed that the pruning of a defined blood vessel CrDI, which locates along the anterior margin of the eye in zebrafish and contains 3–4 ECs, was accompanied by the death of 1–2 ECs (Kochhan et al., 2013), suggesting that the cell apoptosis are caused by the redundancy of ECs. Franco et al. (2015) reported the association of apoptotic events with regression of long vessels in the retina and hypothesized that cell apoptosis are associated with failure of ECs to integrate into adjacent vessels. Consistently, for the developing brain vasculature, we showed that EC apoptosis-accompanied pruned vessels were longer and more likely to have the nuclei of neighboring vessels’ ECs occupying at their both ends. These characteristics may prevent ECs in pruning vessels migrating to and integrating into adjacent vessels, thus leading to EC apoptosis.

In our previous study, we found that vessel pruning in the brain of larval zebrafish occurs preferentially at loop-forming vessels, which usually show inefficient blood flow and are functionally redundant (Chen et al., 2012). Here, EC apoptosis-accompanied vessel pruning also occurred at loop-forming vessels. We speculated that hemodynamic changes in loop-forming vessels will usually trigger the ECs in pruning vessels to migrate and integrate into adjacent unpruned vessels, but if the loop-forming vessels are long in length and occupied by the nuclei of neighboring vessels’ ECs at their both ends, the ECs in pruning vessels may be prevented from migration and instead undergo apoptosis.

## Author Contributions

BX, YZ, JD and XD conceived the project and designed the experiments. BX and YZ carried out the experiments and analyzed the data with QC’s and YY’s help. BX, YZ, JD and XD wrote the manuscript.

## Conflict of Interest Statement

The authors declare that the research was conducted in the absence of any commercial or financial relationships that could be construed as a potential conflict of interest.
